# MR-10 Enhances Men's Health by Improving Endogenous Male Sex Hormone Generation

**DOI:** 10.1089/jmf.2018.4201

**Published:** 2018-12-12

**Authors:** Yoo-Hun Noh

**Affiliations:** ^1^ Department of Anatomy and Cell Biology, College of Medicine, Chung-Ang University, Seoul, Korea.; ^2^ Famenity Biomedical Research Center, Famenity, Inc., Gyeonggi-do, Korea.

**Keywords:** *Korean dandelion*, *men's health*, *MR-10*, *rooibos*, *testosterone*

## Abstract

Although there is a clear need for improving men's health, treatment with suitable natural substances has not yet been well established. Previously, it was reported that MR-10, a novel complex of Korean dandelion and rooibos found by screening many natural products, improved sperm generation and activity. Here, the ability of MR-10 to increase testosterone levels and enhance men's health was tested. Treatment with MR-10 (400 mg/day) for a month significantly increased levels of free testosterone, total testosterone, and the testosterone precursor dehydroepiandrosterone by 22%, 14%, and 32%, respectively, in clinical studies. Also, men's health in terms of mental, physical, and sexual aspects, as determined by using the clinical questionnaires Androgen Deficiency of Aging Men and Aging Males' Symptoms, was improved. Furthermore, the safety of MR-10 was determined by testing levels of prostate-specific antigen, glutamic oxaloacetic transaminase, and glutamic pyruvate transaminase; and the lack of changes due to MR-10 treatment supports the safety of MR-10. In conclusion, this study suggests that MR-10 is a safe and effective natural product improving men's sexual health.

## Introduction

The health of aging men is closely related to the male hormone testosterone. Testosterone deceases by ∼1% per year after the age of 30. The process of aging in men involves various changes in the body, mainly caused by a gradual decline in the levels of testosterone and its metabolic precursor, dehydroepiandrosterone (DHEA).^1^ Despite the fact that DHEA is a precursor of sex hormones, there have been reports of conflicting studies on the correlation with testosterone concentrations. Due to the strong interrelation between steroid hormones, the single assessment of DHEA or testosterone appears to be insufficient to adequately reflect the hormonal state of an individual.^2,3^

Testosterone exists in three forms. Free testosterone (FT) is bioactive, freely diffusing into target cells. A decline in the FT level is a hallmark of andropause, an androgen deficiency syndrome^4–6^ marked by decreased energy and physical activity, depression, decreased libido, erectile dysfunction, decreased muscle mass and strength, increased fat mass, frailty, and osteopenia.^7–9^ Changes in sex hormone-binding globulin (SHBG) do not result in changes in total testosterone (TT) level, whereas a bioavailability, estimated by FT levels, is diminished.^10^ Moreover, andropause increases the risk of developing certain diseases such as ischemic heart disease, diabetes, osteoporosis, prostate cancer, and Alzheimer's disease. It also affects the recovery process after acute illnesses, thus necessitating long-term professional care, which may lead to psychological problems, social isolation, increased mortality, and diminished quality of life.^4,11,12^ Since testosterone replacement therapy with exogenous testosterone has been linked to the development of prostate cancer and cardiovascular disease,^13–15^ it is important to find safe natural products that are beneficial for men's health.

After screening many natural products, we found MR-10, a complex of Korean dandelion and rooibos that improves endogenous sperm generation and activity. Dandelion is an antiviral agent due to its detoxifying effects^16,17^ and also has antibacterial, antifungal, antiviral, antidiabetic, anti-inflammatory, hepatoprotective, diuretic, and tumor apoptosis-inducing properties.^18–20^ Rooibos was first used in the 1700s for its various health promoting properties and can reduce lipid oxidation, oxidative stress, and inflammation, in addition to reverse hyperglycemia and chemically induced liver damage.^21–23^ Previously, we showed that MR-10 protects Leydig cells from age-related stress via the ERK and Akt pathways and enhances endogenous male sex hormone synthesis *in vivo*. In this study, we examined whether MR-10 can enhance the levels of male sex hormones and improve andropause symptoms. Clinical safety of MR-10 was also tested.

## Materials and Methods

### MR-10 and recruitment of participants

MR-10 was prepared as previously described.^24^ In brief, the bioactive components from the Korean dandelion plant *Taraxacum platycarpum* Dahlst. and fermented leaves of the rooibos plant *Aspalathus linearis* (Burm. f.) R. Dahlgren was purified as powder.

Ninety-six male subjects older than 45 years of age were recruited from the Chung-Ang University Medical Center (Seoul, Korea) between January 2012 and May 2016 through a posted advertisement. Men were excluded from the study if they presented with any of the following conditions: metabolic disease, prostatic hyperplasia, prostate cancer, urethral stricture, neurogenic bladder, cardiovascular disease, and/or thyroid disease during the past year and/or prescription use of sex hormones. Patient characteristics are shown in Table 1.

**Table 1.** Demographic Characteristics of Participants

	*MR-10 group (*n* = 48)*			*Placebo group (*n* = 48)*			
	*Before*	*After*	P^ac^	*Before*	*After*	P^ac^	P^ab^*(baseline)*
Age (year)	49.63 ± 1.20		—	50.1 ± 1.43		—	.781
Height (cm)	170.42 ± 0.83		—	170.25 ± 0.84		—	.914
Weight (kg)	72.44 ± 1.39	72.59 ± 1.41	.937	71.82 ± 1.42	71.82 ± 1.41	.998	.862
BMI (kg/m^2^)	24.94 ± 1.39	24.36 ± 1.26	.833	24.71 ± 0.36	24.77 ± 0.36	.909	.917
Total testosterone (ng/mL)	3.50 ± 0.18	3.93 ± 0.23	.009	3.63 ± 0.19	3.59 ± 0.20	.768	.673
Free tesostreone (ng/dL)	0.06 ± 0.003	0.07 ± 0.003	.000	0.06 ± 0.002	0.06 ± 0.002	.793	.991
DHEA (ng/mL)	3.51 ± 0.33	4.23 ± 0.29	.023	3.49 ± 0.42	3.29 ± 0.40	.556	.958
SHBG (nmol/L)	47.29 ± 2.69	43.96 ± 2.60	.002	46.98 ± 2.77	48.33 ± 3.23	.178	.503

Values represent mean ± standard error of mean.

^a^ Two-tailed *P* value.

^b^ Two sample *t*-test, MS-10 versus placebo.

^c^ Paired *t*-test, before versus after.

BMI, body mass index; DHEA, dehydroepiandrosterone; SHBG, sex hormone-binding globulin.

This study was approved by the Medical Ethics Committee of the University of Chung-Ang and performed in accordance with the Helsinki Declaration (No. 2011-10-03). All participants signed an informed consent form. Participants could withdraw from the study at any time, and a serious adverse event was considered an absolute stopping rule.

### Procedure

Demographic and clinical data were obtained through questionnaires and interviews with the participants, and the levels of serum hormones and enzymes were measured. A pharmacist who was blinded to all clinical information randomly distributed each participant to either the MR-10 or placebo (control) group, and each group consisted of 48 participants. The physical characteristics of the placebo, dextran, were identical to those of the test compound. Throughout the study, the investigator, rater, and participants were blinded. Before taking either MR-10 or the placebo, each participant underwent a baseline assessment of height, weight, body mass index (BMI), and serum chemistry. Participants took a capsule containing either 200 mg MR-10 or the placebo twice a day (400 mg/day) for 4 weeks. Participants who failed to take MR-10 or the placebo for more than 2 weeks were excluded from the study analysis. The study's endpoints were the final outcomes and safety measures.

### Questionnaires and parameters

The Androgen Deficiency of Aging Men (ADAM) questionnaire assessed urological and medical histories and the Aging Males' Symptoms (AMS) scale were used at screening, and at weeks 0 and 4. Fasting blood samples were obtained to measure the levels of TT, DHEA, SHBG, FT, prostate-specific antigen (PSA), glutamic oxaloacetic transaminase (GOT), and glutamic pyruvate transaminase (GPT). The levels of TT, DHEA, and SHBG were measured by radioimmunoassay (Diagnostic Products Co., Los Angeles, CA, USA), whereas the FT level was measured according to the method of Vermeulen *et al.*^25^ The serum PSA level was measured using the Elecsys 170 assay (Roche Diagnostics, Mannheim, Germany).

### Statistical analyses

Pearson's chi-squared test and dependent Student's *t*-test were used to compare demographic variables between the MR-10 and control groups. Repeated measures paired *t*-test and ANOVA test was used to identify changes in mean scores of cell viability, height, body weight, BMI, TT, FT, DHEA, SHBG, and AMS scores, before and after the administration of the compounds using covariates such as the intelligence quotient and grade. The McNemar test was used to identify changes in the responses to the ADAM questionnaire, before and after the administration of the compounds. All statistical analyses were performed using IBM SPSS software version 24.0 (IBM Analytics, Armonk, NY, USA). Statistical significance (denoted as *α*) was defined using two-tailed tests, and *P* values less than .05 were considered statistically significant.

## Results

Previously, we found that MR-10 enhances spermatogenesis, sperm activity, and locomotor activity.^24^ A decline in the serum sexual hormone levels in men is the main causative factor leading to andropause. In other words, increasing testosterone levels in aging men improves their health and physical activity. Therefore, to evaluate the positive effects of MR-10 on male sex hormone levels, we measured TT, FT, and DHEA levels in the serum and expressed the results relative to baselines because the personal basal serum levels were highly variable. All of these indexes' blood concentrations before ingestion were statistically similar (*P* > .05).

**FIG. 1.**
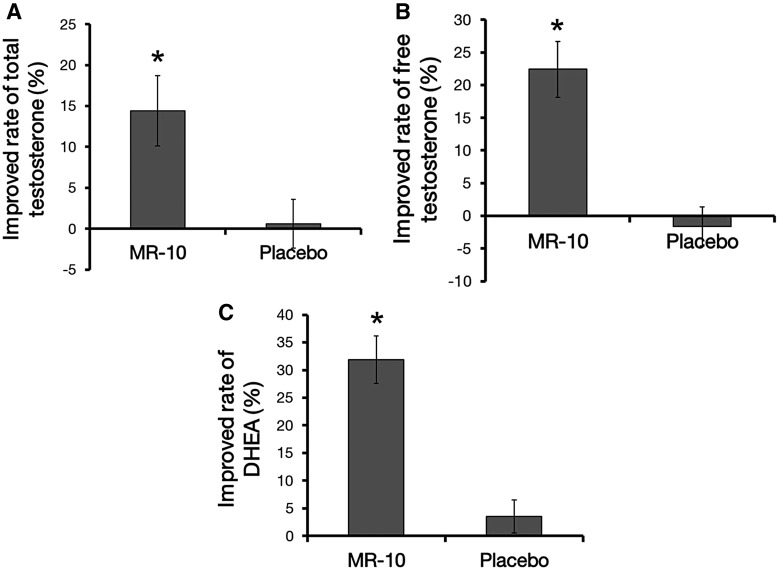
A comparison of serum testosterone, free testosterone, and DHEA levels between MR-10 and placebo groups. **(A)** Change in serum testosterone level in participants treated with MR-10 (*n* = 48) or with the placebo (*n* = 48) (*P < .05 by dependent Student's *t*-test). Solid bars indicate the levels from the MR-10 and placebo groups compared with baseline results. **(B)** Change in free testosterone level in participants treated with MR-10 or placebo (*P < .05 by dependent Student's *t*-test). **(C)** Change in DHEA level in participants treated with MR-10 or placebo (*P < .05 by dependent Student's *t*-test). Data are expressed as percentages. DHEA, dehydroepiandrosterone.

The levels of TT, FT, and DHEA were ∼14.4% (*P* = .010), 22.4% (*P* = .000), and 32% (*P* = .003) higher than basal levels, respectively, after ingestion of MR-10 for 4 weeks, whereas there were no differences in their levels in the placebo control group (Fig. 1A–C).

**FIG. 2.**
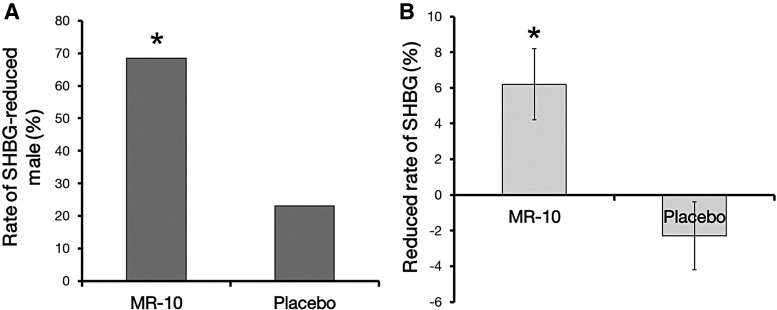
A comparison of SHBG level between MR-10 and placebo groups. **(A)** Proportion of men with reduced SHBG levels in MR-10 (*n* = 48) and placebo (*n* = 48) groups. **(B)** Change in SHBG level in men treated with MR-10 or placebo. (*P < .05 by dependent Student's *t*-test). Data are expressed as percentages. SHBG, sex hormone-binding globulin.

We also measured the SHBG level, since SHBG inactivates testosterone. In the MR-10 treatment group, the level of SHBG was reduced in ∼68% of the men, whereas the number of men in the placebo control group with reduced SHBG levels was not statistically significant (Fig. 2A). SHBG levels were an average of 6.2% (*P* = .003) lower after MR-10 treatment than before treatment (Fig. 2B).

**FIG. 3.**
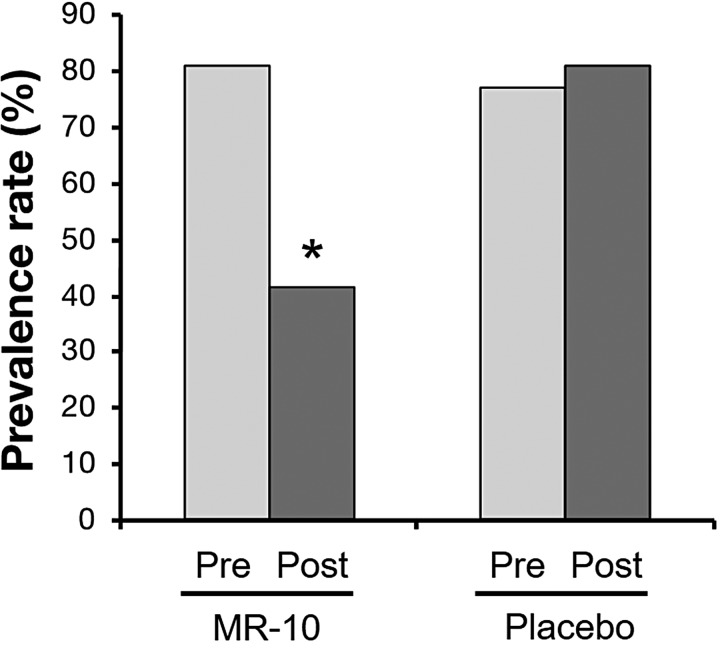
The prevalence of andropause as assessed by the ADAM questionnaire. The ADAM score was determined before and after treatment with 400 mg/day MR-10 for 4 weeks. Data are expressed as a percentage (*P < .05 by Pearson's chi-square test). ADAM, Androgen Deficiency of Aging Men.

We next evaluated whether MR-10 improved the andropause symptoms using clinical questionnaires ADAM and AMS. ADAM can determine what proportion of men that show an improvement. In the MR-10 treatment group, the prevalence of andropause dropped from about 80% to 42% after taking MR-10 for a month at 400 mg/day (Table 2). In the placebo control group, the prevalence was similar before and after treatment (Fig. 3).

**Table 2.** Response to Questionnaires by Androgen Deficiency in the Aging Male Status

	*MR-10 group (*n* = 48)*			*Placebo group (*n* = 48)*			P	
	*Before*	*After*	P^a^	*Before*	*After*	P^a^	*Before*^b^	*After*^b^
Response positive (*n*)	39	20	.000	37	39	.688	.758	.000
Response negative (*n*)	9	28		11	9			

^a^ McNemar tested *P* value, before versus after.

^b^ Pearson's chi-squared tested *P* value, MR-10 versus Placebo.

**FIG. 4.**
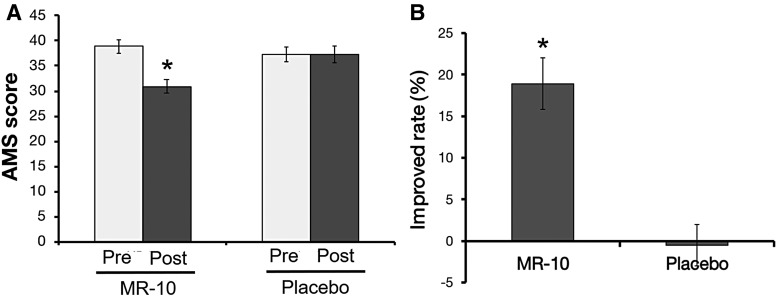
A comparison of the AMS score between MR-10 and placebo groups. **(A)** The AMS score in MR-10 (*n* = 48) and placebo (*n* = 48) groups (*P < .05 by dependent Student's *t*-test). **(B)** Change in AMS score in participants treated with MR-10 or placebo. Adjusted mean difference in the AMS score compared with the baseline AMS score (*P < .05 by dependent Student's *t*-test). AMS, Aging Males' Symptoms.

AMS can quantitatively evaluate andropause symptoms including psychological, somatic, and sexual symptoms.^26^ Therefore, we evaluated in a quantitative manner whether MR-10 improved andropause symptoms. In the MR-10 group, the AMS score changed from 37.2 before treatment to 30.9 after treatment (*P* = .000), whereas no change was observed in the placebo control group (*P* = .981, Fig. 4A). The reduced AMS score represents reduced andropause symptoms. The improvement rate in the MR-10 group was ∼20% (*P* = .000, Fig. 4B). Therefore, MR-10 improved the andropausal symptoms related to male vitality, emotional state, joint and muscle pain, sleep, and sexual function by about 20%.

**FIG. 5.**
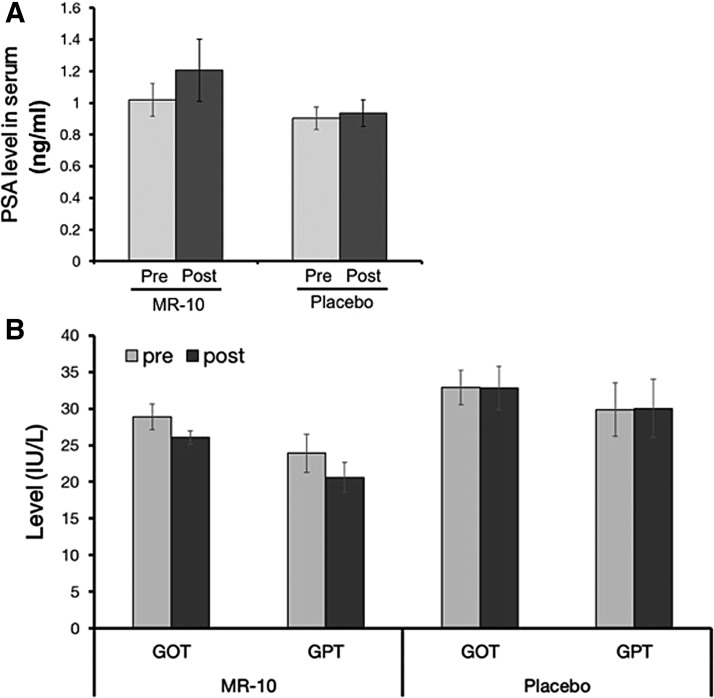
A comparison of serum enzyme levels between MR-10 and placebo groups. **(A)** The PSA level in MR-10 (*n* = 48) and placebo (*n* = 48) groups. **(B)** The GOT and GPT levels in MR-10 and placebo groups. GOT, glutamic oxaloacetic transaminase; GPT, glutamic pyruvate transaminase; PSA, prostate-specific antigen.

We also examined the clinical safety of MR-10 using PSA, a male-specific safety marker, in addition to GOT and GPT, general markers of liver injury. The levels of PSA did not change after treatment in either the MR-10 or placebo group (Fig. 5A). Similarly, neither GOT nor GPT levels changed (Fig. 5B). In summary, our results indicate that MR-10 can upregulate the serum testosterone level, alleviating the symptoms of andropause safely.

## Discussion

Previously, MR-10, dandelion and rooibos extract complex, protected TM3 Leydig cells from typical physiological stress via activation of ERK and Akt pathways.^24^ In a preclinical study using an andropause animal model, the level of testosterone and activation of spermatogenesis were significantly increased and markedly promoted a physical locomotor activity.^24^ In this study, we assessed the putative alleviative effects of MR-10 on the symptoms of andropause through clinical study.

The ADAM and AMS questionnaires evaluate andropause symptoms involved in sexual dysfunction or other symptoms of hormone deficiency in andropause, which has a sensitivity of 88% and a specificity of 60% in men.^27,28^ Our results showed that MR-10 improved symptoms based on the ADAM and AMS scales. Therefore, MR-10 can simultaneously decrease the prevalence of andropause and improve andropausal symptoms, promoting better quality of life for men and improvement of urinary symptoms.

It is well known that a low serum testosterone level is the cause of symptomatic andropause.^29–31^ Increasing the serum testosterone level can improve the symptoms of andropause and the quality of life for aging men.^32,33^ Our results showed that MR-10 increased serum testosterone precursors (DHEA), testosterone, and FT. Therefore, MR-10 could be used to prevent and treat andropause syndrome through upregulation of testosterone and testosterone precursors in the serum.

The SHBG level gradually increases in aging men.^34,35^ Furthermore, the increase in SHBG levels is related to the age-dependent decline in circulating growth hormone or insulin-like growth factor level.^25^ Various chronic diseases (such as diabetes mellitus, coronary atherosclerosis, renal failure, and liver disease) in aging men are associated with decreased (F)T levels and increased SHBG.^36–39^ Therefore, our results indicate that MR-10 has the potential to improve men's health through the cooperative effects of increasing (F)T and decreasing SHBG.

MR-10 also significantly increased the concentration of DHEA, a testosterone precursor with important physiological roles after it is converted into testosterone.^40^ Although it is possible that the increase in testosterone was caused by the increase in DHEA, the increased DHEA level alone alleviated the symptoms of andropause in aging men. DHEA also has various antiaging effects, including reduced inflammation and improved blood flow, bone metabolism, sexual function, and physical strength.^41,42^ In addition, DHEA reduces the symptoms of andropause in aging men.^42,43^

The side effects of testosterone replacement therapy in men with benign prostatic hyperplasia include the development of prostate cancer and cardiovascular disease.^15,33,44^ Studies also report an increase in the PSA level, which can induce prostatic hyperplasia and prostate cancer.^45,46^ In this study, we measured the serum PSA level in men treated with MR-10 and found no difference. Furthermore, there were no differences in GOT and GPT levels. These results clearly indicate the safety of MR-10.

In conclusion, our results show that MR-10 improves the symptoms of andropause by increasing testosterone and DHEA levels, and decreasing SHBG levels, without any adverse side effects. Therefore, MR-10 may be a safe and effective nutraceutical agent for the treatment of andropause symptoms and for improving men's health.
